# Dopamine D1 Receptors Regulate the Light Dependent Development of Retinal Synaptic Responses

**DOI:** 10.1371/journal.pone.0079625

**Published:** 2013-11-19

**Authors:** Quanhua He, Hong-ping Xu, Ping Wang, Ning Tian

**Affiliations:** 1 College of Pharmacy, The Dorothy M. Davis Heart and Lung Research Institute, The Ohio State University, Columbus, Ohio, United States of America; 2 Department of Neurobiology, Yale University School of Medicine, New Haven, Connecticut, United States of America; 3 Department of Ophthalmology and Visual Science, University of Utah School of Medicine, Salt Lake City, Utah, United States of America; Universidade Federal do ABC, Brazil

## Abstract

Retinal synaptic connections and function are developmentally regulated. Retinal synaptic activity plays critical roles in the development of retinal synaptic circuitry. Dopamine receptors have been thought to play important roles in the activity-dependent synaptic plasticity in central nervous system. The primary goal of this study is to determine whether dopamine D1 receptor regulates the activity-dependent development of retinal light responsiveness. Accordingly, we recorded electroretinogram from wild type mice and mice with genetic deletion of D1 dopamine receptor (D1−/− mice) raised under cyclic light conditions and constant darkness. Our results demonstrated that D1−/− mice have reduced amplitudes of all three major components of electroretinogram in adulthood. When the relative strength of the responses is considered, the D1−/− mice have selective reduction of the amplitudes of a-wave and oscillatory potentials evoked by low-intermediate intensities of lights. During postnatal development, D1−/− mice have increased amplitude of b-wave at the time of eye-opening but reduced developmental increase of the amplitude of b-wave after eye opening. Light deprivation from birth significantly reduced the amplitudes of b-wave and oscillatory potentials, increased the outer retinal light response gain and altered the light response kinetics of both a- and b-waves of wild type mice. In D1−/− mice, the effect of dark rearing on the amplitude of oscillatory potentials was diminished and dark rearing induced effects on the response gain of outer retina and the kinetics of a-wave were reversed. These results demonstrated roles of dopamine D1 receptor in the activity-dependent functional development of mouse retina.

## Introduction

Retinal synaptic connections and function are developmentally regulated. These developmental regulations are reflected in changes of density of synapses, expression of neurotransmitter receptors, re-organization of synaptic inputs of different synaptic pathways, excitability and light responsiveness of retinal neurons during development. For example, the light responsiveness of photoreceptors, bipolar cells, and retinal ganglion cells (RGCs) measured using flash and pattern electroretinogram (ERG) of rabbits, lamb, rats, dogs and human increase during postnatal development [1]–[14]. The amplitudes of light responses of retinal ganglion cell (RGC) in cat, ferret and mouse increase after eye opening [15]–[17]. In mouse, the rate of spontaneous synaptic inputs to RGCs increases approximately 4-fold in a period of 1 to 2 weeks after eye opening [17]. In addition, the number of RGCs receiving synaptic inputs from both ON and OFF bipolar cells decreases after eye opening [18]. Visual experience affects the functional and morphological development of retinal synaptic circuitry. Dark rearing increases the density of conventional synapses in the inner plexiform layer (IPL) of mouse and rat retinas [19]–[20] and delayed the bipolar cell morphological maturation in rabbits [21]. In hamster, dark rearing blocked an age-related dendritic modification of “aberrant” RGCs [22]. In mice and rats, dark rearing retarded the maturation of the strength of spontaneous RGC synaptic inputs, the age-dependent segregation of ON and OFF inputs to RGCs, light-evoked responsiveness of RGCs [17]–[18], [23]–[25] and the age-dependent expression of GABA and glutamate receptors of retinal neurons [26]–[29]. Despite the evidence that visual deprivation alters maturational changes in retinal synaptic structures and functions, little is known about the synaptic and molecular mechanisms of activity-dependent developmental regulation of retinal light evoked responses.

Dopamine receptors have been thought to play important roles in the activity-dependent synaptic plasticity in CNS [30]–[33], by regulating the intrinsic excitability of CNS neurons [30], activity of AMPA and NMDA receptors [34]–[40], and GABAergic activity [41]. In retina, dopamine receptors are expressed by all neurons and play important roles for retinal development, synaptic formation and transmission, and adaptations [42]–[43]. For instance, D1 dopamine receptors are expressed by retinal horizontal cells and AII amacrine cells to regulate gap junction connections between these cells [44]–[46]. D1 dopamine receptors are also expressed by RGCs and intrinsic photo-sensitive RGCs (ipRGCs) to regulate light adaptation and light sensitivity of these cells [47]–[48]. In addition, D1 dopamine receptors have been reported to regulate acetylcholine release from amacrine cells [49]–[50], GABA release from horizental cells [51], retinal neuron growth cone motility and neurite outgrowth [52], and activity-dependent control of ocular growth [53]. More interestingly, the expression profile of dopamine receptors or the number of dopaminergic cells during postnatal development indicates that dopamine is involved in retinal development [54]–[55]. In addition, the storage and release of dopamine from dopaminergic amacrine cells is regulated by light and visual activity [55]–[58]. These results raise a possibility that dopamine receptor expression and dopamine receptor mediated signaling might regulate the activity-dependent development of retinal light evoked responses.

The primary goal of this study is to determine whether dopamine D1 receptor regulates the activity-dependent development of retinal light responsiveness. Accordingly, we recorded flash light evoked ERG from mice with genetic deletion of D1 dopamine receptor (D1−/− mice) during postnatal development and compared the results with age-matched wild type (WT) controls. In addition, we examined the ERG responses of both WT and D1−/− mice raised in constant darkness from birth. Our results demonstrated that D1−/− mice have reduced amplitudes of ERG responses, especially the amplitudes of a-wave and OPs evoked by low-intermediate light intensities. At the time of eye-opening, D1−/− mice have increased amplitude of ERG b-wave but reduced developmental increase of the amplitude of ERG b-wave after eye opening. Interestingly, dark rearing of WT mice from birth significantly reduced the amplitudes of b-wave and oscillatory potentials (OPs), increased the outer retinal light response gain to the low light intensity stimulation, and altered the light response kinetics of both a- and b-waves. In D1−/− mice, the effect of dark rearing on the amplitude of OPs was diminished and the dark rearing induced effect on the response gain of outer retina and the changes of the kinetics of ERG a-wave were reversed. These results demonstrated roles of dopamine D1 receptor in the development and activity-dependent functional plasticity of mouse retina.

## Experimental Procedures

### Ethic Statement

All procedures of handling, maintenance and preparation of animals met the NIH guidelines and were approved by the Animal Care and Use Committee of Yale University.

### Animals and dark rearing

ERGs were recorded from both eyes of C57BL/6 (WT) and D1−/− mice (The Jackson Laboratory, Bar Harbor, Maine). Both the WT and D1−/− mice were either raised under cyclic light/dark conditions as controls or under constant darkness. The control animals were fed and housed under 12:12 hour cyclic light/dark conditions in regular mouse rooms located in the Animal Care Facility. The average light intensity illuminating the cages during subjective day was 40 lux for control mice. Dark reared animals were housed in conventional mouse cages, which were placed in a continuously ventilated light-tight box. The temperature and humidity inside the box were continuously monitored and balanced by adjusting the speed of the ventilating fan. The box was placed in a light-tight room located in the same facility as control animals. All the procedures of daily monitoring and routine maintenance of dark reared mice were conducted under infrared illumination by trained personal with the use of a pair of IR sensitive goggles (B.E. Meyers and Co. Inc., Redmond, WA).

### ERG recordings and data analysis

Animals reared under cyclic light/dark conditions were dark-adapted for at least 30 minutes before experiments. Dark reared mice were transferred from dark room to the ERG recording room in a light-tight transfer box. Just prior to the recordings, mice were anesthetized with Xylazine (13 mg/kg) and Ketamine (87 mg/kg) and the pupils were dilated with Atropine (1%, Bausch & Lomb, Pharmaceuticals, Inc., Tampa, FL) and Phenylephrine HCl (Mydfrin 2.5%, Alcon Inc., Humacao, Puerto Rico). A topical anesthetic agent, proparacarine (0.5%, Alcon Inc., Humacao, Puerto Rico) was used before the contact electrodes were applied to the corneas. ERGs were evoked by 100 ms white flashes generated by LED arrays built in to a pair of miniature Ganzfield stimulators for both eyes (EPIC-3000, LKC Technologies Inc., Gaithersburg, MD). Signals were band-pass filtered between 0.3 Hz to 500 Hz. For each of the intensities between 0.008 cd*s/m^2^ (−25 dB) and 0.8 cd*s/m^2^ (−5 dB), ERGs were averaged from 5 single flashes. Inter-stimulus interval was 30 seconds. ERGs were averaged from 3 single flashes for the intensities between 2.5 cd*s/m^2^ (0 dB) and 25 cd*s/m^2^ (10 dB). The inter-stimulus interval was 60 seconds. All recordings were made at approximately the same time of the day.

Responses of ERG components were fitted to the following equation (a modified Naka-Rushton function) using software Igor (WaveMetrics, Lake Oswego, OR) with the Levenberg-Marquardt algorithm to determine the R_max_ and I_50_:
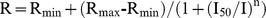
Here R is the amplitude of ERG a-, b-wave or OPs, R_max_ is the amplitude of the saturated responses predicted from the recordings, R_min_ is the amplitude of the minimum responses predicted from the recordings, I is the light intensity for each recorded data point (R), I_50_ is the light intensity at which the half saturated response would be predicted from the recordings (semisaturation constant), and n is a variable that determines the steepness of the curves.

Student t-tests were used to examine the difference between two means. All of the statistical tests were performed using software StatView (Abacus Concepts, Berkeley, CA).

## Results

### The amplitudes of ERG responses are reduced in adult D1−/− mice

**Figure 1:**
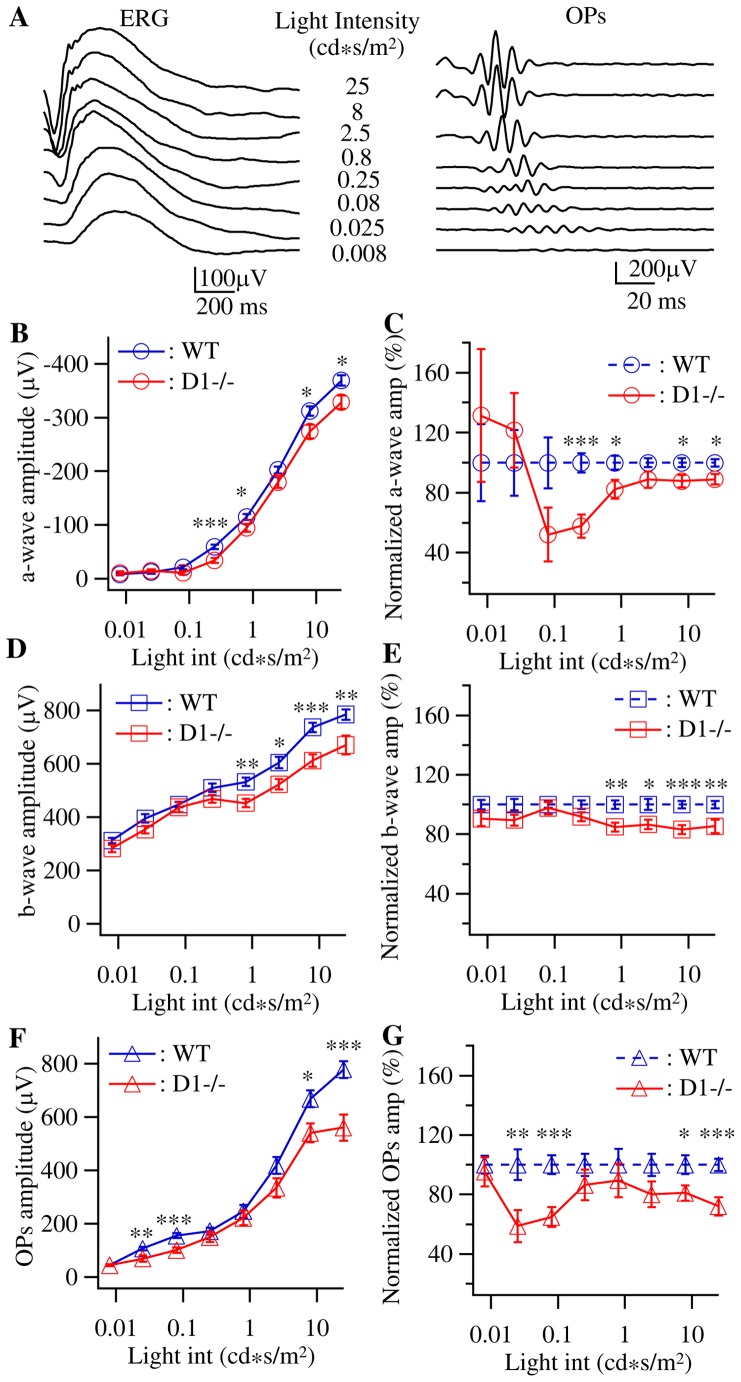
The amplitudes of ERG a-, b-wave and OPs are reduced in D1−/− mice. ERGs were recorded from dark-adapted P30 WT controls and D1−/− mice at eight different intensities of light stimuli. The amplitudes of a-wave, b-wave and OPs were plotted as a function of intensity of light stimuli as intensity-response curve and the amplitudes of ERG a-, b-wave and OPs of D1−/− mice were also normalized to the WT controls to reveal the relative changes of strength of ERG of D1−/− mice. **A:** Representative ERG (left) and OPs (right) waveforms recorded from a P30 WT mouse evoked by 8 different light intensities (from 0.008 cd*s/m^2^ at the bottom to 25 cd*s/m^2^ at the top). **B:** Average intensity-response curves of a-wave amplitude of WT (n = 40 eyes of 20 mice) and D1−/− (n = 20 eyes of 10 mice) mice. **C:** Normalized ERG a-wave shows that the a-wave amplitudes of D1−/− mice were reduced by 40–50% at the light intensities of 0.08–0.25 cd*s/m^2^. **D:** Average intensity-response curves of b-wave amplitude of the same mice as shown in Fig. 1B. **E:** Normalized ERG b-wave showed less than 20% reduction at all light intensities of D1−/− mice. **F:** Average intensity-response curves of OPs amplitude of the same mice as shown in Fig. 1B. **G:** Normalized ERG OPs shows that the OPs amplitudes of D1−/− mice were reduced by 40% at the light intensities of 0.025–0.08 cd*s/m^2^. In all panels, * indicates the difference is statistically significant and p value is between 0.05 and 0.01; ** indicates p value is smaller than 0.01; error bars indicate standard errors in this and all following figures.

The D1 dopamine receptors are found to be expressed in both inner and outer retina [44]–[48] and pharmacologically blockage or mutation of dopamine D1 receptors have been shown to reduce the amplitudes of both a- and b-wave of ERG in rabbit, goldfish and mouse retinas [59]–[62] but enhance the amplitudes of b- and d-wave of both scotopic and photopic ERG in frog retina [63]. We first determine the effects of genetic mutation of dopamine D1 receptor on the ERG of young adult mice. ERGs responding to 8 different light intensities were recorded from D1−/− and WT mice at the age of postnatal day 30 (P30) and the amplitudes of the three major components, a-wave, b-wave and oscillatory potentials (OPs), were plotted as a function of stimulating light intensity to form the intensity-response curves. Fig. 1A shows representative waveforms of ERGs (left) and OPs (right) recorded from a WT mouse. The initial portion of the a-wave is a measurement of photoreceptor function. The b-wave is a measurement of ON bipolar cell function and reflects both photoreceptor and synaptic function in the outer plexiform layer of the retina [64]–[65]. The OPs, which reflects interactions among bipolar, amacrine and ganglion cells, is a measurement of inner retinal function [66]. The OPs shown in Fig. 1A were isolated by bandpass filtering (73 Hz to 500 Hz) the waves of ERGs and the amplitudes of OPs were calculated by the sum of all peaks [67]. The light intensities used to evoke ERG responses cover a wide range from 0.008 to 25 cd*s/m^2^ (−25 log unit to 10 log unit), which will only stimulate rods in the lower intensities but probably both rods and cones at the high intensities. Because no background light was used to isolate cone-mediated light responses, the ERGs recorded in this study are either scotopic or mesopic responses.

Figs. 1B, 1D and 1F show the average intensity-response curves of ERG a-, b-wave and OPs recorded from D1−/− and WT mice, respectively. For a-wave and OPs, the average intensity-response curves of both WT and D1−/− mice have sigmoid distribution, indicating that the light stimuli cover the most of the response dynamic range of photoreceptors and inner retinal neurons. On the other hand, the average intensity-response curves of b-waves of both WT and D1−/− mice have a somewhat linear distribution, indicating that the range of light intensity was probably only wide enough to cover the middle portion of the whole response spectrum of bipolar cells. By comparing the intensity-response curves of a-, b-wave and OPs of D1−/− with WT controls, it is evident that the amplitudes of ERG a-, b-wave and OPs are all decreased in D1−/− mice. This is similar to the effects of D1 receptor antagonists in rabbit retina [59] and a recent report of D1 receptor mutation of mice [62]. Statistic tests (*t*-tests) showed that the differences of the amplitudes of a-, b-wave and OPs between D1−/− and WT mice are significant only for some light intensities mostly towards the high light intensities. We further examined these data by fitting them to a modified Naka-Rushton function (see detailed description in Methods) to predict the maximum a-, b-wave and OPs responses and the hemisaturation constant (I_50_) of D1−/− and WT mice. Consistent with the intensity-response curves, the predicted maximum a-wave and OPs amplitudes are also significantly reduced in D1−/− mice (Table 1). The average maximum a-wave amplitudes are −364.6±16.4 µV versus −445.2±18 µV for D1−/− and WT mice (mean ± SE for these and all following expressions, p = 0.0056, t = 2.88) and the average maximum OPs amplitudes are 653.6±50 µV versus 914.3±44.4 µV for D1−/− and WT mice and (p = 0.0008, t = −3.637). Although the average maximum b-wave amplitude of D1−/− mice is 10.4% lower than that of WT controls (993±88.4 µV versus 1108.1±44 µV), the difference is not statistically significant (p = 0.1963, t = −1.307, Table 1). The I_50_ for a-, b-wave and OPs of D1−/− mice are not statistically different from that of age matched WT controls.

**Table 1:** Predicted maximum ERG responses.

P13	WT a-wave	D1−/− a-wave	WT b-wave	D1−/− b-wave	WT OPs	D1−/− OPs
Mean (µV)	−53.5	−74.2	230.4	254.7	99	106.6
SE (µV)	8.4	10.4	43.7	31	14.6	18.7
n (eyes)	12	11*	12	12	12	12
p and t	0.1332	1.562	0.6538	0.455	0.7514	0.321

P30	WT a-wave	D1−/− a-wave	WT b-wave	D1−/− b-wave	WT OPs	D1−/− OPs
Mean (µV)	−445.2	−364.6	1108.1	992.9	914.3	653.6
SE (µV)	18	16.4	44	88.4	44.4	50
n (eyes)	38*	19*	40	20	39*	20
p and t	0.0056	2.882	0.1963	−1.307	0.0008	−3.637

* : Results of 1–2 eyes of these groups could not be fitted with the model and those eyes are not included.

To further reveal the relative extent of the changes of ERG responses due to D1 receptor mutation, we quantified the differences of the intensity-response curves of ERG a-, b-wave and OPs between D1−/− and WT mice by normalizing the responses of D1−/− mice to the age-matched WT controls using the following equation:
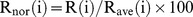
Here R_nor_(i) represents the normalized a-, b-wave or OPs amplitudes of D1−/− mice evoked by light intensity (i). R(i) represents the actual a-, b-wave or OPs amplitudes of D1−/− mice evoked by light stimulus (i). R_ave_(i) is the average amplitudes of a-, b-wave or OPs evoked by light stimulus (i) of WT control mice. Therefore, the results of D1−/− mice are expressed as percentiles of the responses of WT mice. It is interesting to note that although the reduction of the amplitudes of ERG was most significant for responses evoked by high light intensities (Figs. 1B, 1D and 1F), the most noticeable changes of normalized responses are the amplitudes of a-wave and OPs to intermediate light intensities (Figs. 1C and 1G). The normalized a-wave amplitudes were reduced to 52±17.8% and 57.7±7.7% of WT controls for the light intensities of 0.08 cd*s/m^2^ and 0.25 cd*s/m^2^ while the normalized a-wave amplitudes were only reduced to 87.8±4.2% and 89±3.4% of WT controls for the light intensities of 8 cd*s/m^2^ and 25 cd*s/m^2^. Similarly, the normalized OPs amplitudes were reduced to 59±10.7% and 64.8±6.7% of WT controls for the light intensities of 0.025 cd*s/m^2^ and 0.08 cd*s/m^2^ while the normalized OPs amplitudes were reduced to 81±5.2% and 72±6.3% of WT controls for the light intensities of 8 cd*s/m^2^ and 25 cd*s/m^2^. However, the normalized b-wave amplitudes do not show this intensity-dependent reduction (Fig. 1E).

**Figure 2:**
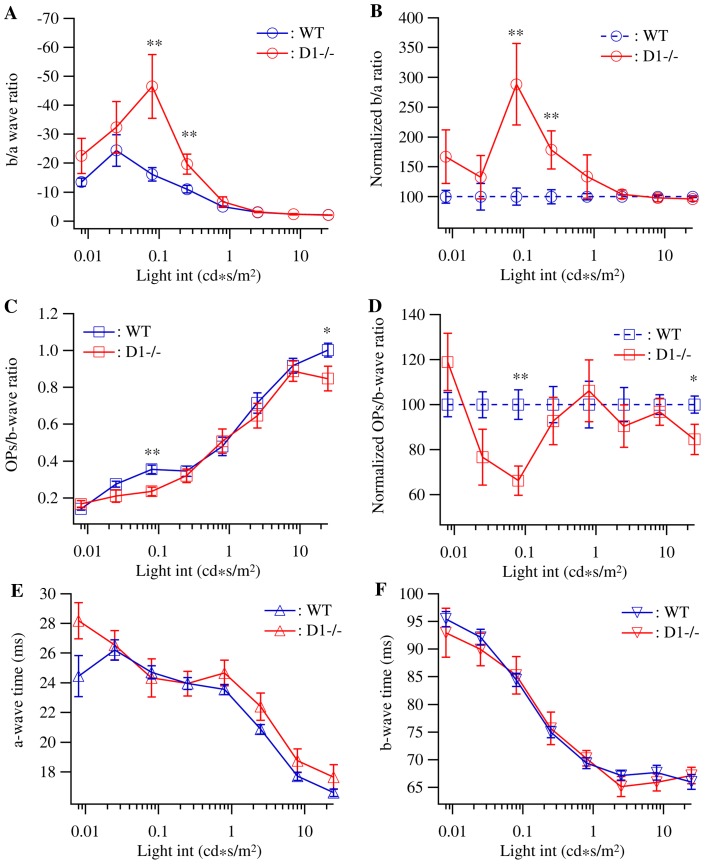
The response gains of inner and outer retina are differentially affected in D1−/− mice. The ratio of b-wave/a-wave was used to assess the response gain of outer retina and the ratio of OPs/b-wave was used to assess the response gain of inner retina. The response gains of D1−/− mice were normalized to that of WT controls to reveal the relative changes of the response gains of D1−/− mice. **A:** The b-wave/a-wave ratios of WT and D1−/− mice plotted as a function of the light intensity showing a significant increase of the response gains at the light intensities of 0.08–0.25 cd*s/m^2^. **B:** The b-wave/a-wave ratios of D1−/− mice normalized to WT controls. **C:** The OPs/b-wave ratios of WT and D1−/− mice plotted as a function of the light intensity. **D:** The OPs/b-wave ratios of D1−/− mice normalized to WT controls showing a significant decrease of the response gains at the light intensities of 0.025–0.08 cd*s/m^2^. **E:** The time to peak of a-wave of WT and D1−/− mice plotted as a function of the light intensity showing no difference between WT and D1−/− mice. **F:** The time to peak of b-wave of WT and D1−/− mice plotted as a function of the light intensity showing no difference between WT and D1−/− mice. The data was from the same groups of WT and D1−/− mice as shown in Fig. 1.

The differences in the changes of the amplitudes of a-, b-wave and OPs of D1−/− mice imply that dopamine D1 receptors might regulate the light responses of inner and outer retina differently through regulating synaptic or response gain between different retinal neurons. To further examine this possibility, we assessed the changes of response gains of outer and inner retina due to D1 receptor mutation by comparing the ratio of b/a-wave and the ratio of OPs/b-wave of D1−/− and WT mice. Although the b/a-wave ratio might not measure the exact synaptic gain between photoreceptors and bipolar cells and the OPs/b-wave ratio might not measure the exact synaptic gain between bipolar cells and RGCs, the changes of these ratios could be used to estimate the changes of the response gains between these neurons. When the average b/a-wave ratios of WT mice were plotted as a function of light intensity (Fig. 2A), the curve is relatively flat with the highest b/a-wave ratio (−24.4±5.45) at the light intensity of 0.025 cd*s/m^2^ and the lowest b/a-wave ratio (−2.14±0.13) at the light intensity of 25 cd*s/m^2^. These results demonstrated that the strength of light response gain at outer retina depends upon the intensity of light stimulation and the light responses evoked by weaker light stimuli have stronger synaptic gain. On the other hand, the D1−/− mice have a much elevated b/a-wave ratio at low-intermediate light intensities. The average b/a-wave ratios of D1−/− mice at the light intensities of 0.08 cd*s/m^2^ and 0.25 cd*s/m^2^ are significantly higher than that of WT controls (−46.5±11 versus −16.1±2.3; and −19.7±3.5 versus −11±1.3 for D1−/− and WT mice; p = 0.0011 and 0.0061, t = 3.592 and 2.849, respectively).

To further reveal the relative extent of the changes of ERG responses gain due to D1 receptor mutation, we normalized the response gain of D1−/− mice to the age-matched WT controls using the following equation:
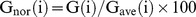
Here G_nor_(i) represents the normalized response gain of D1−/− mice evoked by light intensity (i). G(i) represents the actual response gain of D1−/− mice evoked by light stimulus (i). G_ave_(i) is the average response gain evoked by light stimulus (i) of WT control mice. Therefore, the response gains of D1−/− mice are expressed as percentile of the response gains of WT mice. Fig. 2B shows that the b/a-wave ratios of D1−/− mice at the light intensities of 0.08 cd*s/m^2^ and 0.25 cd*s/m^2^ are 288.4±68.2% and 178.5±31.8% higher than that of WT controls. In contrast, the ratios of OPs/b-wave of D1−/− mice are somewhat overlapped with that of WT controls except at the light intensities of 0.25 cd*s/m^2^ and 25 cd*s/m^2^, where the differences are statistically significant (Fig. 2C, p = 0.0014 and 0.0355, t = 3.363 and 2.153, respectively). The normalized OPs/b-wave ratios of D1−/− mice at the light intensities of 0.25 cd*s/m^2^ and 25 cd*s/m^2^ are 66.2±6.4% and 84.6±6.7% of that of WT controls (Fig. 2D). These results support the notion that dopamine D1 receptor preferentially increases the transmission of visual signaling evoked by low-intermediate light intensities in outer retina but decreases the transmission of visual signaling evoked by low-intermediate light intensities in the inner retina. Furthermore, we examined the light response kinetic of ERG by measuring the peak times of ERG a-wave (Fig. 2E) and b-wave (Fig. 2F) of both D1−/− and WT mice. We found no significant difference of the peak time for both a- and b-wave between WT and D1−/− mice.

Overall, the results shown above demonstrated a preferential reduction of light response of retinal neurons located at both inner and outer retina evoked by high light intensity and some notable reduction of amplitudes of a-wave and OPs evoked by intermediate light intensities. Although the magnitude of the amplitude changes of a-wave and OPs evoked by intermediate light intensities is not as significant as the changes of a-wave and OPs evoked by high light intensities, the relative impact could be stronger because the overall response evoked by intermediate light intensities is much smaller.

### Mutation of dopamine D1 receptor reduces the developmental increase of ERG b-wave amplitudes

**Figure 3:**
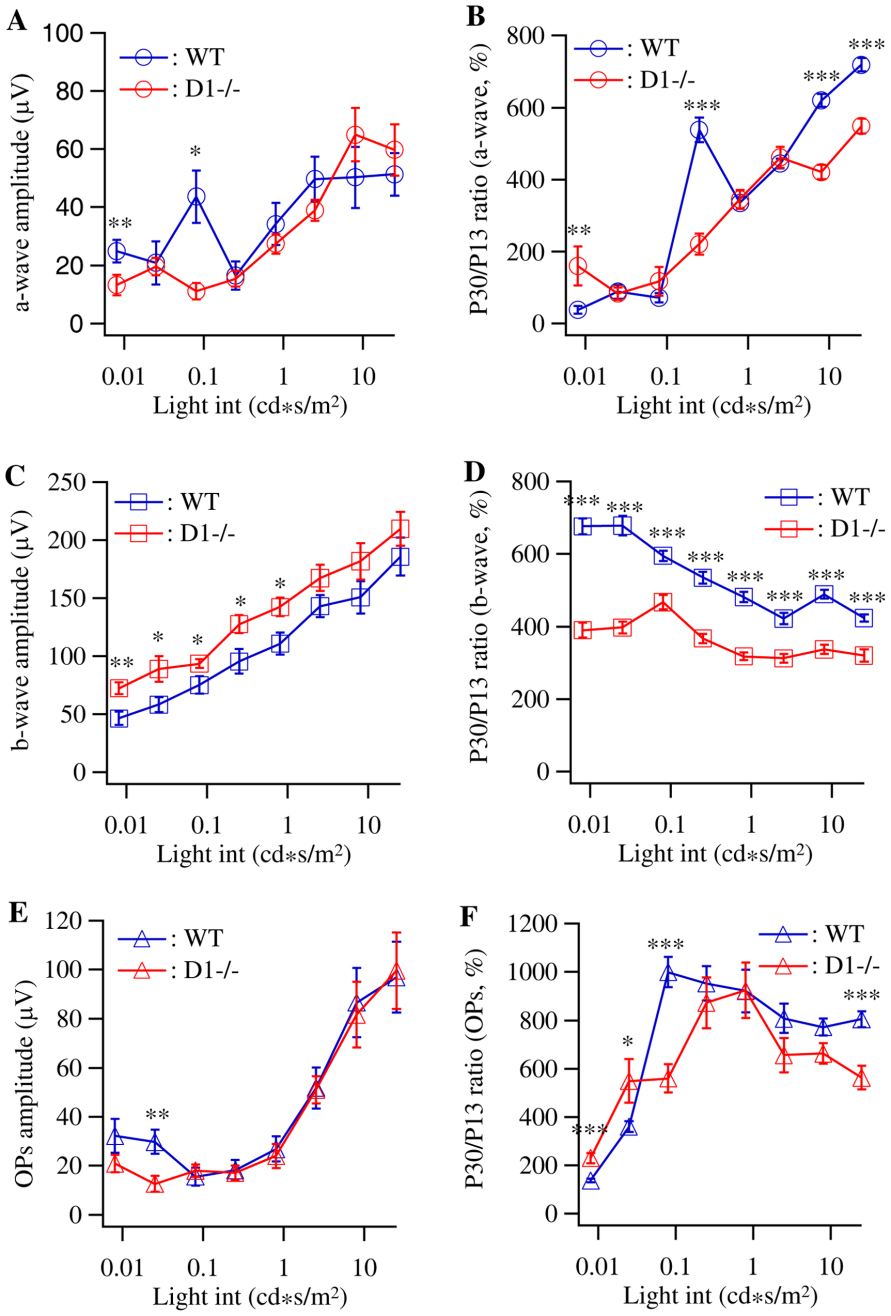
D1−/− mice have selective increase of ERG b-wave amplitude at the time of eye opening. ERGs were recorded from dark-adapted WT and D1−/− mice at the age of P13. The amplitudes of a-wave, b-wave and OPs were plotted as a function of intensity of light stimuli and the amplitudes of ERG a-wave, b-wave and OPs of P13 WT and D1−/− mice were compared with that of P30 mice to reveal the developmental changes of ERG amplitudes. **A:** Average intensity-response curves of a-wave amplitude of WT (n = 12 eyes of 6 mice) and D1−/− (n = 12 eyes of 6 mice) mice. **B:** The P30/P13 ratios of ERG a-wave amplitudes of WT and D1−/− mice as a function of light intensity. **C:** Average intensity-response curves of b-wave amplitude of the same groups WT and D1−/− mice. **D:** The P30/P13 ratios of b-wave amplitudes of WT and D1−/− mice as a function of light intensity. **E:** Average intensity-response curves of OPs of WT and D1−/− mice. **F:** The P30/P13 ratios of OPs amplitudes of WT and D1−/− mice as a function of light intensity.

It has been shown that the amplitudes of ERG undergo significant developmental enhancement during postnatal development in human and other mammals [1]–[2], [4], [6], [10]–[11], [13], [68]–[70]. To determine whether mutation of dopamine D1 receptor impacts the development of light responsiveness of retina, we recorded ERGs from WT and D1−/− mice at the time of eye opening (P13) and compared with the ERGs of P30 WT and D1−/− mice. Figs. 3A, 3C and 3E show the average intensity-response curves of ERG a-, b-wave and OPs recorded from D1−/− and WT mice at the age of P13, respectively. Similar to the P30 mice, the average intensity-response curves of a-wave and OPs of both WT and D1−/− mice have sigmoid distribution, while the average intensity-response curves of b-waves of both WT and D1−/− mice have a linear distribution. The average amplitudes of ERG a-wave and OPs of P13 D1−/− mice are not significantly different from that of age-matched WT controls in most light intensities (Figs. 3A and 3E) except at 1–2 light intensities. Interestingly, the average amplitudes of ERG b-wave of P13 D1−/− mice are higher than that of age-matched WT controls by 56.7% at 0.008 cd*s/m^2^ and 13% at 25 cd*s/m^2^. The differences are statistically significant for the 5 low-intermediate light intensities (0.008 cd*s/m^2^ to 0.8 cd*s/m^2^) but not the 3 high light intensities (2.5 cd*s/m^2^ to 25 cd*s/m^2^) (Fig. 3C). Although the predicted maximum amplitudes of a-, b-wave and OPs of D1−/− mice are increased in comparison with the age-matched WT controls (Table 1), the differences between the predicted maximum values of WT and D1−/− mice are not statistically significant (Table 1). One might argue that the lack of statistical significance of the predicted maximum a-wave amplitudes between WT and D1−/− mice is due to big variation of data or insufficient number of mice tested. Nonetheless, these results support the idea that mutation of D1 receptor has minimum effects on the light responsiveness of inner retinal neurons at the early stage of synaptic development of retina but selectively enhances bipolar cell light responses.

We then determined whether mutation of dopamine D1 receptor affects the developmental changes of ERG responses. We first calculated the ratios of ERGs recorded at P13 and P30 to determine the magnitude of developmental changes of the major components of ERGs of WT and D1−/− mice and then compared the developmental changes of D1−/− mice with WT controls. Fig. 3B shows the P30/P13 ratios of ERG a-wave amplitudes of WT and D1−/− mice as a function of light intensity. Although with significant variation, it is evident that the P30/P13 ratios of a-wave amplitudes are greater than 100% at most light intensities for both WT and D1−/− mice except a few intensities (from 38.9±10% and 160.4±53.9% at 0.008 cd*s/m^2^ and 0.025 cd*s/m^2^ to 718.4±18.7% and 548.5±21% at 25 cd*s/m^2^ for WT and D1−/− mice, respectively). In addition, the value of the P30/P13 ratio increases with the light intensity for both WT and D1−/− mice. Furthermore, the P30/P13 ratios of ERG a-wave amplitudes of WT and D1−/− mice have a similar distribution pattern at most light intensities and the distribution curves are not systematically different except a few sporadic points of light intensities where either WT or D1−/− mice have higher ratio. These results demonstrated that the a-wave amplitudes increase from P13 to P30 for both WT and D1−/− mice and mutation of dopamine D1 receptor has limited effect on the maturation of photoreceptor light response.

Similar to the P30/P13 ratios of ERG a-wave, the P30/P13 ratios of ERG b-wave are also greater than 100% at all light intensities for both WT and D1−/− mice (from 676.1±21.2% and 389.9±20.6% at 0.008 cd*s/m^2^ to 423.1±10.6% and 319.7±17% at 25 cd*s/m^2^ for WT and D1−/− mice, respectively) (Fig. 3D), demonstrating that the amplitudes of b-wave increased by approximately 4–7 fold from P13 to P30 for both WT and D1−/− mice. However, the distributions of P30/P13 ratios of ERG b-wave amplitudes are significantly different from the P30/P13 ratios of a-wave in the following three aspects. First, the value of the P30/P13 ratio of b-wave decreased, but not increased, with the light intensity in WT mice from 676.1±21.2% at the light intensity of 0.008 cd*s/m^2^ to 423.1±10.6% at the light intensity 25 cd*s/m^2^. Second, the P30/P13 ratios of b-wave amplitudes of D1−/− mice are significantly lower than that of WT controls at all light intensities. Third, the difference of the P30/P13 ratio of b-wave amplitudes between WT and D1−/− mice at low light intensities is much bigger than that at high light intensities (WT versus D1−/− mice; 676.1±21.2% versus 389.9±20.6% at 0.008 cd*s/m^2^; 423.1±10.6% versus 319.7±17% at 25 cd*s/m^2^) and, therefore, the preference of the developmental increase of rod-mediated b-wave amplitudes observed in WT mice is diminished in D1−/− mice. These results demonstrated that dopamine D1 receptor is required for the developmental increase of bipolar cell light responses and D1 receptors seem to have a stronger effect on the maturation of rod bipolar cell light responses.

Fig. 3F shows the P30/P13 ratios of OPs amplitudes of WT and D1−/− mice as a function of light intensity. Again, the P30/P13 ratios of OPs amplitudes of WT and D1−/− mice are greater than 100% at all light intensities (from 136.9±7.9% and 203.4±20.5% at 0.008 cd*s/m^2^ to 804±32.9% and 563.2±49.2% at 25 cd*s/m^2^ for WT and D1−/− mice, respectively). Different from the distributions of P30/P13 ratios of both a- and b-wave, the distributions of the P30/P13 ratios of OPs amplitudes of WT and D1−/− mice show a clear biphasic pattern. The P30/P13 ratios increase with light at the low intensities (0.008 cd*s/m^2^ to 0.08 cd*s/m^2^) and the ratio curve remains relatively flat at intermediate-high light intensities (0.25 cd*s/m^2^ to 25 cd*s/m^2^). Similar to the P30/P13 ratios of ERG a-wave, the P30/P13 ratios of OPs amplitudes of WT and D1−/− mice have similar distribution curves, which roughly overlapped at most light intensities with a few sporadic points of light intensities where either WT or D1−/− mice have higher ratio, suggesting that dopamine D1 receptors play a limited role to the maturation of OPs.

### Mutation of dopamine D1 receptor selectively blocks the effects of light deprivation on the amplitudes of OPs

**Figure 4:**
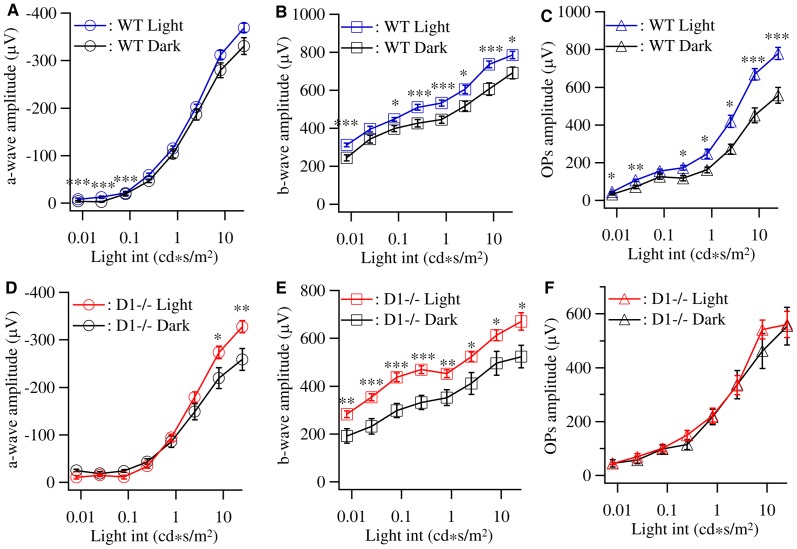
Light deprivation suppresses the OPs amplitudes of WT but not D1−/− mice. To determine the effects of light deprivation on ERG amplitudes, WT and D1−/− mice were raised in constant darkness from birth and the ERGs were recorded at P30. **A:** Average intensity-response curves of a-wave amplitude of WT mice raised in cyclic light/dark conditions (WT Light, 20 mice, 40 eyes) and constant darkness (WT Dark, 7 mice, 14 eyes). **B:** Average intensity-response curves of b-wave amplitude of WT mice raised in cyclic light/dark conditions and constant darkness. **C:** Average intensity-response curves of OPs of WT mice raised in cyclic light/dark conditions and constant darkness showing significant decrease of the OPs amplitudes of dark-reared mice. **D:** Average intensity-response curves of a-wave amplitude of D1−/− mice raised in cyclic light/dark conditions (D1−/− Light, 10 mice, 20 eyes) and constant darkness (D1−/− Dark, 6 mice, 12 eyes). **E:** Average intensity-response curves of b-wave amplitude of D1−/− mice raised in cyclic light/dark conditions and constant darkness. **F:** Average intensity-response curves of OPs of D1−/− mice raised in cyclic light/dark conditions and constant darkness showing no significant difference between the OPs amplitudes of dark-reared mice and mice raised under cyclic light/dark conditions.

The developmental enhancement of ERG responses of mouse retina has been shown to be sensitive to light deprivation. Long-term light deprivation of developing vertebrates significantly altered the amplitudes of all three major components of flash light ERG [15], [25], [71]–[74]. Because dopamine D1 receptor has been shown to participate in the activity-dependent synaptic plasticity in CNS [30]–[33], we investigated whether dopamine D1 receptor participates in the activity-dependent development of ERG response of mice. We dark reared both D1−/− and WT mice from birth to P30 and compared the ERG responses of these dark reared mice with that of age-matched controls raised under cyclic light conditions. Similar to previous reports, WT mice raised in constant darkness from birth to P30 have reduced amplitudes of all three major components of ERG, especially the amplitudes of OPs for high light intensities. The amplitudes of a-waves were reduced to 46.8±20.9% and 89.7±4.8% of control levels at light intensities of 0.008 cd*s/m^2^ and 25 cd*s/m^2^, respectively (Fig. 4A). The amplitudes of b-waves were reduced to 78.3±5.4% and 88±3.9% of control levels at light intensities of 0.008 cd*s/m^2^ and 25 cd*s/m^2^, respectively (Fig. 4B), and the amplitudes of OPs were reduced to 71.6±6.8% and 71.5±5.4% of control levels at light intensities of 0.008 cd*s/m^2^ and 25 cd*s/m^2^, respectively (Fig. 4C). Similar to the WT mice, the amplitudes of a- and b-waves of dark reared D1−/− mice are reduced as that of dark reared WT mice (Figs. 4D and 4E). However, the amplitudes of OPs of D1−/− mice raised in constant darkness are not different from that of age-matched D1−/− mice raised under cyclic light conditions at all light intensities (Fig. 4F). These results suggest that light stimulation differentially regulate the response gains in the outer and inner retina through dopamine D1 receptor-mediated signaling.

### Mutation of dopamine D1 receptor differentially affects the response gain and the kinetics of inner and outer retina of dark reared mice

**Figure 5:**
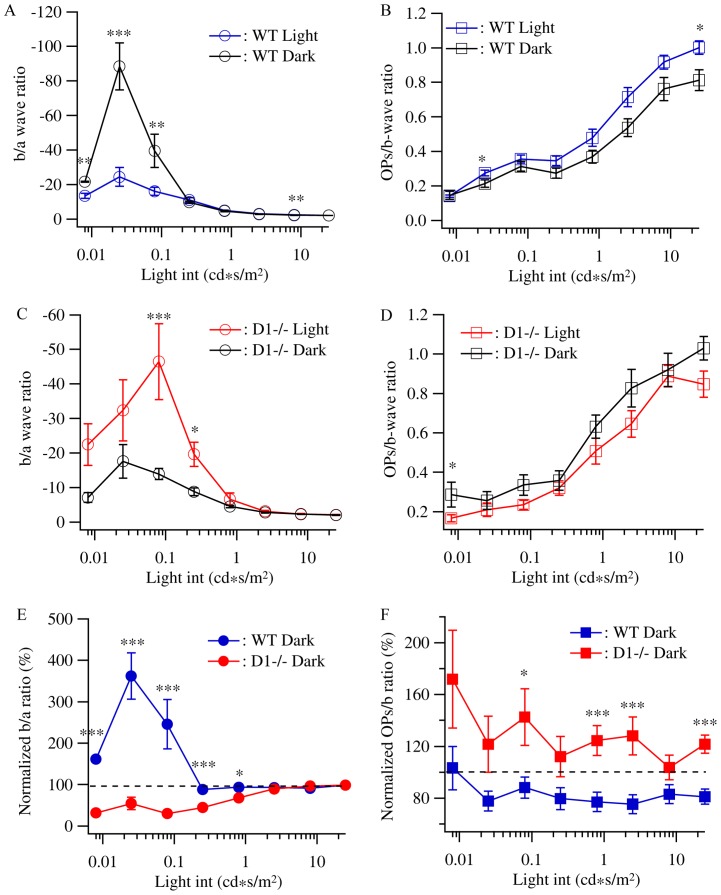
Dopamine D1 receptors mediate the light-sensitive response gain of outer retina. The b-wave/a-wave ratios and OPs/b-waves ratios of WT and D1−/− mice raised under cyclic light/dark conditions and constant darkness were used to assess the effects of light deprivation on the response gains of ERG. **A:** The b-wave/a-wave ratios of WT mice raised under cyclic light/dark conditions and constant darkness were plotted as a function of the light intensity showing a significant increase of the outer retina response gain at the light intensities of 0.008–0.08 cd*s/m^2^. **B:** The OPs/b-wave ratios of WT mice raised under cyclic light/dark conditions and constant darkness. **C:** The b-wave/a-wave ratios of D1−/− mice raised under cyclic light/dark conditions and constant darkness showing a significant decrease of the outer retina response gain at the light intensities of 0.08–0.25 cd*s/m^2^. **D:** The OPs/b-wave ratios of D1−/− mice raised under cyclic light/dark conditions and constant darkness. **E:** The b-wave/a-wave ratios of D1−/− mice raised in constant dark were normalized to D1−/− mice raised in cyclic light/dark conditions while the b-wave/a-wave ratios of WT mice raised in constant dark were normalized to WT mice raised in cyclic light/dark conditions. The two normalized b-wave/a-wave curves were plotted as functions of light intensities and showed that light deprivation significantly increased the b-wave/a-wave ratios of light responses evoked by low light intensities in WT but not D1−/− mice. **F:** The OPs/b-wave ratios of D1−/− and WT mice raised in constant dark were normalized to control mice raised in cyclic light/dark conditions, respectively, and plotted as functions of light intensities.

To further test this possibility, we analyzed the b/a-wave ratio and the OPs/b-wave ratio of WT and D1−/− mice raised in constant darkness and compared with that of D1−/− and WT mice raised under cyclic light conditions. Fig. 5A shows that the b/a-wave ratio of WT mice raised under cyclic light conditions is relatively flat as a function of light intensity. The b/a-wave ratios of dark reared WT mice at low light intensities were significantly increased while the b/a-wave ratios at light intensities higher than 0.25 cd*s/m^2^ were not different from that of WT control mice. The most significant effect was observed at the light intensity of 0.025 cd*s/m^2^, where the b/a-wave ratio of dark reared WT mice (−88.4±13.7) was 3.7-fold higher than that of WT control mice (−24.4±5.5). On the other hand, the D1−/− control mice have significantly higher b/a-wave ratios at low-intermediate light intensities than that of high light intensities. Instead of increasing the b/a-wave ratio as dark-reared WT mice, dark rearing significantly decreased the b/a-wave ratios at low-intermediate light intensities of D1−/− mice. The b/a-wave ratio of D1−/− control mice at 0.08 cd*s/m^2^ was −46.5±11 and it was decreased by 70% (13.9±1.6) in dark reared D1−/− mice (Fig. 5C, p = 0.0078, t = 2.929). Similar to WT mice, the b/a-wave ratios of D1−/− mice for the high light intensities are not affected by dark rearing. Fig. 5E compares the effects of dark rearing on the b/a-wave ratios of WT and D1−/− mice. The b/a-wave ratios of dark reared WT mice are normalized to the age-matched WT controls and the b/a-wave ratios of dark reared D1−/− mice are normalized to the age-matched D1−/− control mice. It is evident that both dark rearing and D1 receptor mutation preferentially affect the b/a-wave ratios at low-intermediate light intensities and the dark rearing has opposite effects on b/a-wave ratios of WT and D1−/− mice.

In contrast to the effects on the b/a-wave ratio, dark rearing has very little effect on the OPs/b-wave ratio of both WT and D1−/− mice (Figs. 5B and 5D). In addition, dark rearing reduced, but not increased, the OPs/b-wave ratio of WT mice and slightly increased, but not reduced, the OPs/b-wave ratio of D1−/− mice. By comparing the normalized OPs/b-wave ratios of dark reared WT and D1−/− mice, it is evident that dark rearing slightly decreased the OPs/b-wave ratio in WT mice and slightly increased the OPs/b-wave ratio in D1−/− mice in a similar magnitude for all light intensities (Fig. 5F).

**Figure 6:**
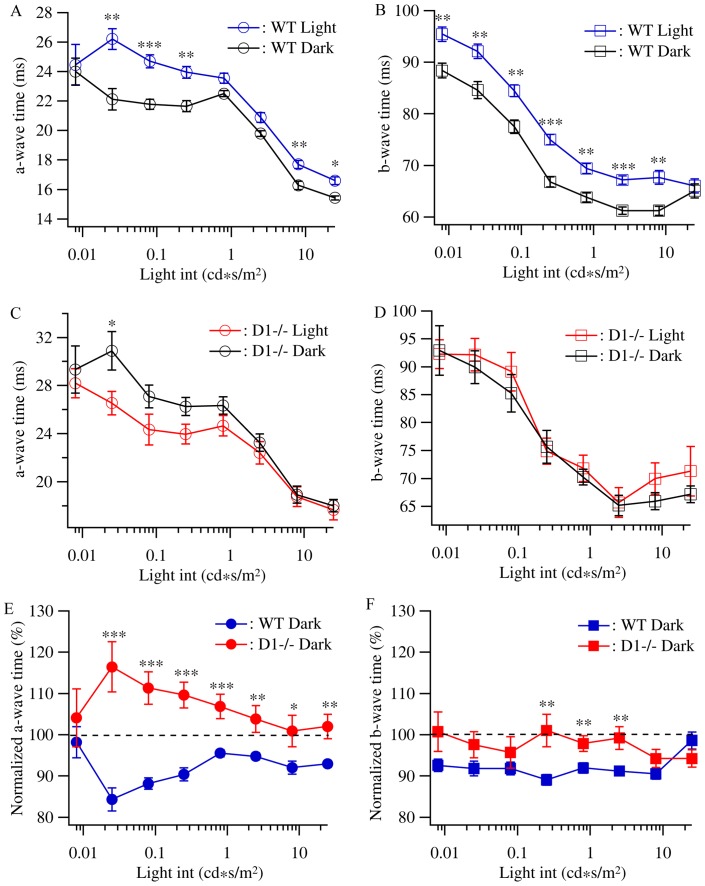
Dopamine D1 receptors mediate the light-sensitive kinetics of ERG a-wave and b-wave. The time to peak of ERG a-wave and b-wave of WT and D1−/− mice raised under cyclic light/dark conditions and constant darkness were measured and plotted as a function of the light intensity. **A:** The time to peak of a-wave of WT mice raised under cyclic light/dark conditions and constant darkness plotted as a function of the light intensity. **B:** The time to peak of b-wave of WT mice raised under cyclic light/dark conditions and constant darkness plotted as a function of the light intensity. **C:** The time to peak of a-wave of D1−/− mice raised under cyclic light/dark conditions and constant darkness plotted as a function of the light intensity. **D:** The time to peak of b-wave of D1−/− mice raised under cyclic light/dark conditions and constant darkness plotted as a function of the light intensity. **E:** The time to peak of ERG a-wave of D1−/− and WT mice raised in constant dark were normalized to that of D1−/− and WT control mice raised in cyclic light/dark conditions, respectively, and plotted as functions of light intensities, showing that light deprivation had opposite effect on the time to peak of ERG a-wave of WT and D1−/− mice. **F:** The time to peak of ERG b-wave of D1−/− and WT mice raised in constant dark were normalized to control mice raised in cyclic light/dark conditions, respectively, and plotted as functions of light intensities.

We further examined the effects induced by dark rearing on the light response kinetics of ERG by measuring the peak times of ERG a-wave and b-wave of both WT and D1−/− mice raised in constant darkness and cyclic light conditions. For WT mice, dark rearing reduced the peak time of both a- and b-waves and the differences between dark reared and control WT mice are statistically significant at most of the light intensities (Figs. 6A and 6B). For D1−/− mice, dark rearing slightly increased the a-wave peak time while most of the differences are statistically insignificant (Fig. 6C). Dark rearing had little effect on the b-wave peak time (Fig. 6D). Figs. 6E and 6F compare the effects of dark rearing on the a- and b-wave peak times of WT and D1−/− mice by normalizing the a- and b-wave peak times of dark reared mice to the genetic- and age-matched controls. Interestingly, dark rearing induced effects on a-wave peak times of both WT and D1−/− mice showed a light intensity dependency. Similar to the b/a-wave ratio, the changes of the a-wave peak times in dark reared mice have a higher magnitude for responses evoked by low-intermediate light intensities except the lowest intensity (Fig. 6E). On the other hand, dark rearing had much weaker effect on the b-wave peak time of both WT and D1−/− mice and the changes across all the light intensities have the similar magnitude (Fig. 6F).

Overall, these results demonstrated that mutation of dopamine D1 receptor selectively eliminated the effects of dark rearing on the OPs amplitude without effect on dark rearing induced reduction of the amplitudes of a- and b-wave. In addition, mutation of dopamine D1 receptor reversed the effects of dark rearing on the outer retinal response gain measured as the b/a-wave ratio. Furthermore, mutation of dopamine D1 receptor reversed the effect of dark rearing on the photoreceptor response kinetics from decreasing the a-wave peak time in WT mice to increasing the a-wave peak time in D1−/− mice preferentially for low-intermediate light intensities.

## Discussion

There are three fundamental findings in this study. First, mutation of dopamine D1 receptor reduced the amplitudes of all three major ERG components, a-, b-wave and OPs, evoked by high intensity of light in adult mice. When the relative strength of the ERG responses are considered, the D1−/− mice have selective reduction of the amplitudes of a-wave and OPs evoked by low-intermediate intensities of lights. Second, dopamine D1 receptor selectively regulates the development of ON bipolar cell light response. At early stage of the synaptic development of retina, mutation of D1 receptor selectively increases the amplitudes of ON bipolar cell light responses measured as ERG b-wave. However, mutation of D1 receptor selectively reduced the developmental increase of the amplitude of ERG b-wave during late postnatal development after eye opening. Third, dark rearing of WT mice significantly reduced the amplitudes of b-wave and OPs, increased the outer retinal light response gain to the low intensity of light, and altered the light response kinetics of both a- and b-waves. Mutation of D1 receptor diminished the effects of dark rearing on the amplitudes of OPs, reversed the dark rearing induced effects on the response gain of outer retina and the changes of the kinetics of ERG a-wave. These results demonstrated roles of dopamine D1 receptor in the activity-dependent functional development of mouse retina.

### The effects of D1 receptor on ERG of adult mice

The effects of D1 dopamine receptor on the ERG seem to be contradictory. It has been reported that mice with genetic deletion of D1 dopamine receptors have reduced amplitudes of b-wave of both scotopic and photopic ERG evoked by flash light with intensities comparable to ours [51], [61]–[62]. Consistently, D1 receptor agonist, SKF 38393, greatly enhanced the amplitudes of b-wave, while D1 receptor antagonist, SCH 23390, reduced the amplitudes of b-wave of light adapted ERG in goldfish [60]. In human, blocking of D1 dopamine receptors reduced the amplitudes of both b-wave and OPs [75]. However, both D1 agonists and antagonists provoked a reduction of the amplitudes of b-wave of scotopic and photopic ERG evoked by 0.1 cd*s/m^2^ and 3 cd*s/m^2^ of lights in rabbits [59]. In frogs, pharmacological blockage of D1 dopamine receptors increased the amplitudes of b- and d-waves of both dark and light adapted ERG with more significant effect to the rod-mediated responses [63], while D1 dopamine receptors antagonists increased the retinal ganglion cell responses in cats [76]. In rabbit and mudpuppy retinas, selective depletion of retinal dopamine resulted in pronounced increase of the amplitudes of ERG a-, b-wave and OPs [77]–[78]. Blockade of dopamine uptake at low concentration enhance the amplitudes of OPs but reduce the amplitudes of OPs at high concentration in rabbits [79].

Our results of D1−/− mice at P30 showed reduced amplitudes of all three major ERG components, a-, b-wave and OPs. The decreasing of the amplitudes of ERG a- and b-wave is consistent with the recent reports of ERG of D1−/− mice by others [51], [61]–[62]. It is worth to note that the D1−/− mice have selective reduction of the relative strength of a-wave and OPs evoked by low-intermediate intensities of lights. This is consistent with the idea that dopamine D1 receptors preferentially regulate rod-dominated visual signals. Although the reduction of the amplitude of OPs with blockade or mutation of D1 dopamine receptors has not been reported previously, it was reported that dopamine regulates the amplitudes of OPs in two opposite directions in a concentration dependent manner. *In vivo* application of a dopamine uptake blocker, nomifensine, at low concentration enhanced the amplitude of OPs in rabbits but attenuated the amplitude of OPs at high concentration [78]. These results imply that dopamine might regulate OPs through two different subtypes of dopamine receptors and activation of D1 receptors needs high concentration of dopamine.

Interestingly, the selective reduction of the relative strength of a-wave and OPs evoked by low-intermediate intensities of lights resemble the response profiles of light mediated gap junction coupling between horizontal cells and AII amacrine cells. It has been well demonstrated that both horizontal cells and AII amacrine cells form gap junctions with neighboring horizontal and AII amacrine cells and the conductance of these gap junctions is regulated by light in a triphasic manner [80]. In fully dark adapted retina, the conductance of gap junctions between horizontal cells and AII amacrine cells is low and, therefore, each horizontal cell or AII amacrine cell only couples with a few neighboring horizontal or AII amacrine cells. Under background light as dim as 0.25 to 1.5 log units above rod threshold, the extent of tracer coupling between both horizontal cells and AII amacrine cells increases by 7–25 times. However, further increase of background light will uncouple the horizontal and AII amacrine cells [81]–[84]. Therefore, coupling is maximized under dim light conditions and diminishes as the retina is dark or light adapted. Because both horizontal cells and AII amacrine cells express D1 dopamine receptors [44]–[46], D1 receptor-mediated signaling regulates gap junction coupling of these cells [85]–[89], and light regulates the activity of D1 dopamine receptor through controlling the release of dopamine in retina [55]–[58], this light-dependent triphasic regulation of gap junction coupling between horizontal cells and AII amacrine cells is considered as a D1 dopamine receptor-mediated adaptation mechanism to enhance retinal sensitivity to light under dim ambient illuminate conditions [80]. Consistently, mutation of the gene for the gap junction protein of AII-AII amacrine cell coupling, CX36, resulted in decreases of the light sensitivity of the most sensitive RGCs in mice [90]. The triphasic changes of horizontal and AII amacrine cell gap junction coupling are very similar to the changes of the response curves of the relative strength of a-wave and OPs we observed from D1−/− mice. Therefore, it is plausible to assume that the selective reduction of the relative strength of OPs evoked by low-intermediate intensities of lights in D1−/− mice might be the result of the lack of light induced increase of AII-AII amacrine cell coupling through activation of D1 dopamine receptors. However, whether and how the change of gap junction conductance of horizontal cells alters the light responses of rods and the relative strength of ERG a-wave need to be further determined.

An unexpected finding of this study is that D1−/− mice showed opposite changes on the response gains of outer and inner retina. In the outer retina, the ratio of b/a-wave is enhanced in D1−/− mice, especially for the response evoked by low intensities of lights, while in the inner retina, the ratio of OPs/b-wave is decreased at the similar light intensity. Although the amplitudes of all the three ERG components are reduced in D1−/− mice, the magnitude of the reduction of b-wave is significantly smaller than that of a-wave and OPs, especially the responses evoked by low intensities of lights, which resulted in the changes of the response gains of outer and inner retina in opposite directions. Because D1 receptor is not expressed by photoreceptors or bipolar cells but only horizontal cells in the outer retina, the changes of response gain in the outer retina must be regulated by horizontal cells. Numerous studies have shown that activation of D1 dopamine receptors on horizontal cells could reduce gap junction connections between these cells, increase trans-membrane calcium currents, increase GABA release from horizontal cells to rod bipolar cells to sensitize the bipolar cells, and regulate the synaptic structures of these cells [46], [51], [80], [85]–[86], [91]–[93]. The change of response gain between photoreceptors and bipolar cells could be the results of either a D1 receptor-mediated developmental synaptic alteration or a D1 receptor-mediated adaptation mechanism. A more specific study is needed to further distinguish these two possibilities. In the inner retina, D1 receptor is expressed by both amacrine and ganglion cells [44]–[45], [47]–[48], [87]–[89] and both of these two types of cells could participate in the generation of OPs [69]–[70]. In addition to the lack of D1-receptor mediated AII-AII amacrine cell coupling, further investigation of whether D1 dopamine receptors regulate synaptic transmission between bipolar, amacrine and ganglion cells will help to have a better understanding of how D1 dopamine receptors regulate visual signal processing in the inner retina.

### The effects of D1 receptor on ERG of developing mice

The changes of ERG during postnatal development have been widely reported and the ages at which ERG parameters reach adult values vary considerably across species. In human, the a- and b-waves are present at birth. The amplitudes of these waves increase considerably during postnatal development and reach the adult level by the ages of 3–15 years [2], [4], [13], [68], indicating an immediate capability of light responsiveness after birth but a continuous maturation period of 15 years for human retina. However, the amplitude ratio a-/b-wave remains constant during postnatal development [4], suggesting that the response gain at outer retina is balanced and stabilized during the whole course of the postnatal development. On the other hand, the OPs are the most immature component of ERG in early infancy, but develop quickly and reach the adult level by two years of age [13], implying a much faster development process than that of ERG a- and b-wave and a possible early increase and later decrease of response gain at inner retina. In guinea pig, the amplitudes of ERG detected at birth was 50% of the adult level and reached maximal values 12 days after birth [94]. In rabbit and rat, the initiation of retinal light responsiveness is later and the maturation process takes much longer time than that of guinea pig. The a-wave appears during the second postnatal week and the amplitude of a-wave reaches the adult value by the age of P30–40. After the a-wave, the b-wave and OPs appear and rapidly grow between the second and third weeks but continues to increase slowly after P40 [3], [5]–[6], [10], [70]–[71], [95]–[98]. Therefore, the developmental profiles of retina vary significantly among different species of rodents. The increase of ERG amplitudes in postnatal development seems related to the maturation of retinal neurons [94], [99]–[100] and no study has reported whether dopamine receptors play roles on the postnatal development of retina.

Similar to rabbits and rats, ERG responses can be detected from mice at the age of P10 in our laboratory (data not shown). After eye opening, the amplitudes of all three major ERG components are increased from P13 to P30 in mice. Interestingly, we noticed that the age-dependent increase of ERG amplitudes is light intensity-dependent and the light intensity-dependency of a-, b-wave and OPs have significant different patterns. The photoreceptor light responses measured as ERG a-wave have a much weaker age-dependent enhancement at low intensity of light but much stronger age-dependent enhancement at high intensity of light. Because the retinal responses to minimum and maximum light intensities could be used to evaluate the retinal threshold sensitivity and maximum capability to light stimulation, these results suggest that the threshold sensitivity matures earlier and is less affected during postnatal development than the maximum light response capability at the photoreceptor level. On the other hand, the bipolar cell light responses measured as ERG b-wave have much stronger age-dependent enhancement at low intensity of light but much weaker age-dependent enhancement at high intensity of light, indicating that the maturation of retina has a stronger effect on bipolar cell threshold sensitivity than the maximum light response capability although both are enhanced during postnatal development. Because the threshold sensitivity of bipolar cells increased much more than that of photoreceptors, the developmental enhancement of bipolar cell threshold sensitivity must be the result of the enhancement of synaptic gain between photoreceptor and bipolar cells. The P30/P13 ratios of OPs, a measurement of the inner retinal responses, have a biphasic pattern with increasing of the ratio at low intensity of lights but without intensity-dependent increase at high intensities of light, supporting the idea that the maturation of retina has stronger effect on the threshold sensitivity of inner retina for intermediate intensities of light. Therefore, our results demonstrated age-dependent changes of retinal light responsiveness at all three different cellular levels during postnatal development and suggested that the developmental enhancements of the retinal light responsiveness at different cellular levels are likely to be regulated by different mechanisms.

One goal of this study is to address the question of whether D1 dopamine receptors regulate the development of retinal light responsiveness. Our results demonstrated that D1 dopamine receptors selectively regulate the postnatal development of bipolar cell light responses. Although the amplitudes of all the three major components of the ERG of D1−/− mice increased with age, they are all reduced in comparison with age-matched WT controls. In contrast to the adult mice, D1−/− mice at P13 showed an increase, but not a decrease, of the amplitude of ERG b-wave, indicating that D1 receptor-mediated signaling regulates the ERG b-wave in a bidirectional manner. This bidirectional regulation appears to be inhibitory during early postnatal development but excitatory in adulthood. Therefore, genetic mutation of D1 dopamine receptor will release the inhibition and enhance b-wave amplitude at early postnatal development but reduce the excitation and decrease b-wave amplitude in adults. Such bidirectional regulation could be achieved by D1 dopamine receptor mediated GABA release from horizontal cells. It was shown that activation of D1 dopamine receptors on horizontal cells promotes GABA release from these cells and the GABA released from horizontal cells can sensitize rod-bipolar cells to enhance rod-bipolar cell light responses by activating GABAc receptors on these cells in adult mice [51]. It is possible that the GABA released from horizontal cells might activate GABAa receptors on rod-bipolar cells in immature retinas and, therefore, suppress rod-bipolar cell light response in young mice. However, this possibility needs to be further investigated by determining the developmental profile of GABAa receptor-mediated synaptic response of rod bipolar cells, especially the responses on the dendritic terminals. In addition, the preferential enhancement of threshold sensitivity of bipolar cells during postnatal development is diminished in D1−/− mice, suggesting a preferential defect of rod bipolar cell dominated response with D1 receptor mutation. If this developmental enhancement of the threshold sensitivity of the rod-dominated bipolar cell response represents the changes of dopamine D1 receptor-regulated GABAc receptor activation on bipolar cells [51], this signaling pathway must undergo a developmental enhancement after eye opening. Furthermore, the significant change of ERG b-wave in developing retina with D1 dopamine receptor mutation seems to have little effect on the amplitude of OPs, suggesting an additional compensatory mechanism between bipolar cell light response and inner retinal light response. Taken together, these results demonstrated that D1 dopamine receptor is required for the maturation of bipolar cell light responses and support the idea that D1 receptors inhibit the bipolar cell light responses at young mice and this inhibition might be important for the development of bipolar cell light responses, especially the rod-dominated bipolar cell responses.

### The role of D1 dopamine receptor on the activity-dependent development of ERG

Another goal of this study is to determine whether D1 dopamine receptors participate in the light deprivation induced defects of the development of retinal light responsiveness. The effects of light deprivation on ERG have been reported previously. Dark rearing or monocular light deprivation of cats for 2 to 4 weeks significantly suppressed b-wave amplitudes [72]–[73]. Dark rearing mice from birth to P30–90 significantly decreased the amplitudes of ERG a-, b-waves and OPs. Interestingly, light deprivation induced suppression of the amplitudes of OPs can be completely reversed by returning the mice back to cyclic light/dark conditions for 1 to 2 weeks. However, the recovery time course was age-dependent with younger animals needing a longer time to achieve a full recovery, indicating that the effects induced by light deprivation on the ERG are more likely due to developmental or activity-dependent plasticity but not a simple adaptation [25].

Our current study confirmed previous observations that mice raised under constant darkness have reduced amplitudes of ERG a-, b-waves and OPs. Because the amplitudes of ERG b-wave are reduced by dark rearing in both WT and D1−/− mice, it strongly suggests that light deprivation does not suppress the amplitudes of ERG b-wave through D1 dopamine receptor-mediated mechanism. Although it has been shown that dark rearing delayed the bipolar cell morphological maturation in rabbits during early postnatal development, most bipolar cells reach mature morphology by the time of eye opening (P10–11) [21]. Therefore, it is unlikely that the reduced ERG b-wave of dark reared WT and D1−/− mice at P30 is the result of delayed morphological maturation of bipolar cells. Interestingly, dark rearing increased the b-/a-wave ratio of WT mice but decreased the b-/a-wave ratio of D1−/− mice. The opposite effects of dark rearing on the b-/a-wave ratio of WT and D1−/− mice seem to be the result of a stronger reduction of b-wave of dark reared D1−/− mice, particularly the b-wave evoked by low intensity of lights. Because dopamine release in the retina is likely to be reduced in dark reared animals [56]–[58], [101]–[104], dark rearing would reduce the activation of D1 dopamine receptors in WT mice but not induce any D1 receptor mediated effects in D1−/− mice. The fact that dark rearing reduced the b-/a-wave ratio of D1−/− mice to the level of WT mice raised under the cyclic light conditions suggests that dopamine reduces the b-/a-wave ratio through activation of D1 dopamine receptors and enhances the b-/a-wave ratio through another mechanism. Without D1 dopamine receptors in D1−/− mice, the b-/a-wave ratio was enhanced and the enhancement of the b-/a-wave ratio was completely diminished by light deprivation. Possibly, dopamine enhances the b-/a-wave ratio through D2/D4 family receptors. Consistent with this idea, pharmacologically activation of D2 dopamine receptors enhanced the amplitude of ERG b-wave [105] while genetic disruption of gene for D4 dopamine receptors significantly reduced the amplitude of ERG b-wave [106].

In the inner retina, the amplitude of OPs is reduced in D1−/− mice raised under cyclic light conditions, indicating that D1 receptor-mediated activity enhance the amplitude of OPs. However, dark rearing of WT mice reduced the amplitudes of OPs to the same level as that of D1−/− mice raised under cyclic light conditions and dark rearing of D1−/− mice had no additional effect on the amplitudes of OPs although the amplitude of b-wave of D1−/− mice is significantly reduced. Even though WT and D1−/− mice raised in constant darkness both have reduced amplitudes of a- and b-waves, the lack of effectiveness of light deprivation on the amplitudes of OPs of D1−/− mice strongly supports the idea that the activity-dependent regulation of inner retinal light responses is dominated by the D1 dopamine receptors.

Light deprivation has been shown to regulate the development of several important cellular and synaptic features in the inner retina, such as the expression of BDNF, GABA and glutamate receptors of retinal neurons [27]–[30], [107], the formation of the inhibitory synapses in the inner plexiform layer (IPL) of mouse and rat retinas [19]–[20], the dendritic refinement of RGCs of hamster and mouse retinas [18], [22], [108], the strength of spontaneous RGC synaptic inputs [17] and the size of inhibitory receptive field of RGCs [109]. However, others have reported that visual deprivation has no discernible effect on retinal light-evoked responses as assessed by changes in the amplitude of RGC responses to light [110]–[113]. It is increasingly clear that bipolar cells, amacrine cells and RGCs are all heterogeneous not only in structure and function but also in the underlying regulatory mechanism for their maturation and, possibly responses to light deprivation. A better understanding of how light deprivation and D1 dopamine receptors regulate the amplitude of OPs will critically depend upon the more precise identification of the exact cellular and synaptic origins of the OPs.

Another interesting question is whether the defects observed from the D1−/− mice are the results of activity-dependent synaptic plasticity or merely a disruption of adaptation/circadian rhythms. It is clear that the postnatal development of retina involves both structural and functional changes and light deprivation causes both temporal and long-term changes of retinal structure and function [1]–[29]. Dopamine receptors certainly have the capability of regulating the structural and functional changes of synapses of neurons of both CNS and retina [30–43]. Interestingly, a recent study showed that dopamine D1 receptors only regulate the baseline amplitude but not the rhythmic changes of ERG b-wave and dopamine D1 receptor agonist, SKF38393, or L-DOPA could rescue the defects of visual acuity and circadian rhythms of ERG but not the amplitude of ERG of dopamine-deficient mice [61]. Taken together with our earlier finding that the light deprivation induced suppression of the amplitudes of OPs can be completely reversed by returning the mice back to cyclic light/dark conditions for 1 to 2 weeks [25], it is unlikely that the effect of dark rearing or dopamine D1 receptor mutation on the amplitude of OPs is the results of a simple adaptation or an alteration of circadian rhythms. However, whether D1 dopamine receptor regulates additional structural synaptic plasticity with light deprivation needs to be further investigated.

## References

[1] Ben-Shlomo G, Ofri R (2006) Development of inner retinal function, evidenced by the pattern electroretinogram, in the rat. Exp Eye Res 83: 417–423. 16626702 10.1016/j.exer.2006.01.020

[2] Breton ME, Quinn GE, Schueller AW (1995) Development of electroretinogram and rod phototransduction response in human infants. Invest Ophthal Vis Sci 36: 1588–1602. 7601640

[3] el-Azazi M, Wachtmeister L (1990) The postnatal development of the oscillatory potentials of the electroretinogram. I. Basic characteristics. Acta Ophthalmol (Copenh) 68: 401–409. 2220355 10.1111/j.1755-3768.1990.tb01667.x

[4] Flores-Guevara R, Renault F, Ostré C, Richard P (1996) Maturation of the electroretinogram in children: stability of the amplitude ratio a/b. Electroencephalogr Clin Neurophysiol 100: 422–427. 8893659

[5] Gorfinkel J, Lachapelle P (1990) Maturation of the photopic b-wave and oscillatory potentials of the electroretinogram in the neonatal rabbit. Can J Ophthalmol. 25: 138–144. 2361195

[6] Gorfinkel J, Lachapelle P, Molotchnikoff S (1988) Maturation of the electroretinogram of the neonatal rabbit. Doc Ophthalmol 69: 237–245. 3168725 10.1007/BF00154404

[7] Hamilton R, Dudgeon J, Bradnam MS, Mactier H (2005) Development of the electroretinogram between 30 and 50 weeks after conception. Early Hum Dev 81: 461–464. 15935923 10.1016/j.earlhumdev.2004.10.019

[8] Kirk GR, Boyer SF (1973) Maturation of the electroretinogram in the dog. Exp Neurol 38: 252–264. 4690181 10.1016/0014-4886(73)90149-0

[9] Parness-Yossifon R, Mets MB (2008) The electroretinogram in children. Curr Opin Ophthalmol 19: 398–402. 18772672 10.1097/ICU.0b013e32830abf11

[10] Reuter JH (1976) The development of the electroretinogram in normal and light-deprived rabbits. Pflugers Arch 363: 7–13. 944912 10.1007/BF00587395

[11] Sandalon S, Ofri R (2012) Age-related changes in the pattern electroretinogram of normal and glatiramer acetate-immunized rats. Invest Ophthalmol Vis Sci 53: 6532–6540. 22918635 10.1167/iovs.12-10103

[12] Zhou X, Huang X, Chen H, Zhao P (2010) Comparison of electroretinogram between healthy preterm and term infants. Doc Ophthalmol 121: 205–213. 20878205 10.1007/s10633-010-9248-8

[13] Westall CA, Panton CM, Levin AV (1998–1999) Time courses for maturation of electroretinogram responses from infancy to adulthood. Doc Ophthalmol 96: 355–379. 10.1023/a:1001856911730 10855811

[14] Woods JR Jr, Parisi V, Coppes V, Brooks DE (1983) Maturational sequence of the visual system: serial measurements of visual evoked potential and electroretinogram in the healthy neonatal lamb. Am J Obstet Gynecol 145: 738–743. 6829663 10.1016/0002-9378(83)90583-5

[15] Tian N, Copenhagen DR (2001) Visual deprivation alters development of synaptic function in inner retina after eye opening. Neuron 32: 439–449. 11709155 10.1016/s0896-6273(01)00470-6

[16] Tootle JS (1993) Early postnatal development of visual function in ganglion cells of the cat retina. J Neurophysiol 69: 1645–1660. 8509831 10.1152/jn.1993.69.5.1645

[17] Wang GY, Liets LC, Chalupa LM (2001) Unique functional properties of on and off pathways in the developing mammalian retina. J Neurosci 21: 4310–4317. 11404416 10.1523/JNEUROSCI.21-12-04310.2001 PMC6762748

[18] Tian N, Copenhagen DR (2003) Visual stimulation is required for refinement of ON and OFF pathways in postnatal retina. Neuron 39: 85–96. 12848934 10.1016/s0896-6273(03)00389-1

[19] Fisher LJ (1979) Development of retinal synaptic arrays in the inner plexiform layer of dark-reared mice. J Embryol Expt Morphol 54: 219–227. 528867

[20] Sosula L, Glow PH (1971) Increase in number of synapses in the inner plexiform layer of light deprived rat retinae: quantitative electron microscopy. J Comp Neurol 141: 427–451. 4101678 10.1002/cne.901410403

[21] Wu ML, Chiao CC (2007) Light deprivation delays morphological differentiation of bipolar cells in the rabbit retina. Brain Res 1170: 13–19. 17716634 10.1016/j.brainres.2007.06.091

[22] Wingate RJ, Thompson ID (1994) Targeting and activity-related dendritic modification in mammalian retinal ganglion cells. J Neurosci 14(11 Pt 1): 6621–6637. 10.1523/JNEUROSCI.14-11-06621.1994 PMC6577235 7965065

[23] Fujikado T, Hosohata J, Omoto T (1996) ERG of form deprivation myopia and drug induced ametropia in chicks. Cur Eye Res 15: 79–86. 10.3109/02713689609017614 8631207

[24] Giovannelli A, Di Marco S, Maccarone R, Bisti S (2008) Long-term dark rearing induces permanent reorganization in retinal circuitry. Biochem Biophys Res Commun 365: 349–354. 17999915 10.1016/j.bbrc.2007.10.204

[25] Vistamehr S, Tian N (2004) Light deprivation suppresses the light response of inner retina in both young and adult mouse. Vis Neurosci 21: 23–37. 15137579 10.1017/s0952523804041033

[26] Guenther E, Schmid S, Wheeler-Schilling T, Albach G, Gründer T, et al (2004) Developmental plasticity of NMDA receptor function in the retina and the influence of light. FASEB J 18: 1433–1435. 15247153 10.1096/fj.03-0618fje

[27] Lee EJ, Gibo TL, Grzywacz NM (2006) Dark-rearing-induced reduction of GABA and GAD and prevention of the effect by BDNF in the mouse retina. Eur J Neurosci 24: 2118–2134. 17074038 10.1111/j.1460-9568.2006.05078.x

[28] Xue J, Li G, Laabich A, Cooper NG (2001) Visual-mediated regulation of retinal CaMKII and its GluR1 substrate is age-dependent. Brain Res Mol Brain Res 93: 95–104. 11532343 10.1016/s0169-328x(01)00168-1

[29] Xue J, Cooper NG (2001) The modification of NMDA receptors by visual experience in the rat retina is age dependent. Brain Res Mol Brain Res 91: 196–203. 11457510 10.1016/s0169-328x(01)00141-3

[30] Edelmann E, Lessmann V (2013) Dopamine regulates intrinsic excitability thereby gating successful induction of spike timing-dependent plasticity in CA1 of the hippocampus. Front Neurosci 7: 25. 23508132 10.3389/fnins.2013.00025 PMC3589711

[31] Surmeier DJ, Ding J, Day M, Wang Z, Shen W (2007) D1 and D2 dopamine-receptor modulation of striatal glutamatergic signaling in striatal medium spiny neurons. Trends Neurosci 30: 228–235. 17408758 10.1016/j.tins.2007.03.008

[32] Surmeier DJ, Shen W, Day M, Gertler T, Chan S, et al (2010) The role of dopamine in modulating the structure and function of striatal circuits. Prog Brain Res 183: 149–167. 20696319 10.1016/S0079-6123(10)83008-0 PMC4431764

[33] Surmeier DJ, Carrillo-Reid L, Bargas J (2011) Dopaminergic modulation of striatal neurons, circuits, and assemblies. Neuroscience 198: 3–18. 21906660 10.1016/j.neuroscience.2011.08.051 PMC3235731

[34] Chergui K (2011) Dopamine induces a GluN2A-dependent form of long-term depression of NMDA synaptic responses in the nucleus accumbens. Neuropharmacol 60: 975–981. 10.1016/j.neuropharm.2011.01.047 21295045

[35] Herwerth M, Jensen V, Novak M, Konopka W, Hvalby O, et al (2012) D4 dopamine receptors modulate NR2B NMDA receptors and LTP in stratum oriens of hippocampal CA1. Cereb Cortex 22: 1786–1798. 21955919 10.1093/cercor/bhr275

[36] Smith WB, Starck SR, Roberts RW, Schuman EM (2005) Dopaminergic stimulation of local protein synthesis enhances surface expression of GluR1 and synaptic transmission in hippocampal neurons. Neuron 45: 765–779. 15748851 10.1016/j.neuron.2005.01.015

[37] Sun X, Zhao Y, Wolf ME (2005) Dopamine receptor stimulation modulates AMPA receptor synaptic insertion in prefrontal cortex neurons. J Neurosci 25: 7342–7351. 16093384 10.1523/JNEUROSCI.4603-04.2005 PMC6725299

[38] Wolf ME (2010) Regulation of AMPA receptor trafficking in the nucleus accumbens by dopamine and cocaine. Neurotox Res 18: 393–409. 20361291 10.1007/s12640-010-9176-0 PMC3935242

[39] Xing B, Kong H, Meng X, Wei SG, Xu M, et al (2010) Dopamine D1 but not D3 receptor is critical for spatial learning and related signaling in the hippocampus. Neuroscience 169: 1511–1519. 20600656 10.1016/j.neuroscience.2010.06.034

[40] Zhu G, Huang Y, Chen Y, Zhuang Y, Behnisch T (2012) MPTP modulates hippocampal synaptic transmission and activity-dependent synaptic plasticity via dopamine receptors. J Neurochem 122: 582–593. 22651101 10.1111/j.1471-4159.2012.07815.x

[41] Xu TX, Yao WD (2010) D1 and D2 dopamine receptors in separate circuits cooperate to drive associative long-term potentiation in the prefrontal cortex. Proc Natl Acad Sci U S A 107: 16366–16371. 20805489 10.1073/pnas.1004108107 PMC2941310

[42] Dowling JE (2012) The retina. Cambridge: The Belknap Press of Harvard University Press.

[43] Nguyen-Legros J, Versaux-Botteri C, Vernier P (1999) Dopamine receptor localization in the mammalian retina. Mol Neurobiol 19: 181–204. 10495103 10.1007/BF02821713

[44] Kothmann WW, Massey SC, O'Brien J (2009) Dopamine-stimulated dephosphorylation of connexin 36 mediates AII amacrine cell uncoupling. J Neurosci 29: 14903–14911. 19940186 10.1523/JNEUROSCI.3436-09.2009 PMC2839935

[45] Mills SL, Xia XB, Hoshi H, Firth SI, Rice ME, et al (2007) Dopaminergic modulation of tracer coupling in a ganglion-amacrine cell network. Vis Neurosci 24: 593–608. 17711603 10.1017/S0952523807070575 PMC2213423

[46] Zhang AJ, Jacoby R, Wu SM (2011) Light- and dopamine-regulated receptive field plasticity in primate horizontal cells. J Comp Neurol 519: 2125–2134. 21452210 10.1002/cne.22604 PMC3632401

[47] Van Hook MJ, Wong KY, Berson DM (2012) Dopaminergic modulation of ganglion-cell photoreceptors in rat. Eur J Neurosci 35: 507–518. 22304466 10.1111/j.1460-9568.2011.07975.x PMC3339262

[48] Vaquero CF, Pignatelli A, Partida GJ, Ishida AT (2001) A dopamine- and protein kinase A-dependent mechanism for network adaptation in retinal ganglion cells. J Neurosci 21: 8624–8635. 11606650 10.1523/JNEUROSCI.21-21-08624.2001 PMC3245881

[49] Hensler JG, Dubocovich ML (1986) D1-dopamine receptor activation mediates [3H] acetylcholine release from rabbit retina. Brain Res 398: 407–412. 2948615 10.1016/0006-8993(86)91506-4

[50] Hensler JG, Cotterell DJ, Dubocovich ML (1987) Pharmacological and biochemical characterization of the D-1 dopamine receptor mediating acetylcholine release in rabbit retina. J Pharmacol Exp Ther 243: 857–867. 2961878

[51] Herrmann R, Heflin SJ, Hammond T, Lee B, Wang J, et al (2011) Rod vision is controlled by dopamine-dependent sensitization of rod bipolar cells by GABA. Neuron 72: 101–110. 21982372 10.1016/j.neuron.2011.07.030 PMC3197016

[52] Lankford K, De Mello FG, Klein WL (1987) A transient embryonic dopamine receptor inhibits growth cone motility and neurite outgrowth in a subset of avian retina neurons. Neurosci Lett 75: 169–174. 2952906 10.1016/0304-3940(87)90292-8

[53] Stone RA, Lin T, Iuvone PM, Laties AM (1990) Postnatal control of ocular growth: dopaminergic mechanisms. Ciba Found Symp 155: 45–57. 2088680 10.1002/9780470514023.ch4

[54] Klitten LL, Rath MF, Coon SL, Kim JS, Klein DC, et al (2008) Localization and regulation of dopamine receptor D4 expression in the adult and developing rat retina. Exp Eye Res 87: 471–477. 18778704 10.1016/j.exer.2008.08.004 PMC2597030

[55] Melamed E, Frucht Y, Vidauri J, Uzzan A, Rosenthal J (1986) Effect of postnatal light deprivation on the ontogenesis of dopamine neurons in rat retina. Brain Res 391: 280–284. 3008950 10.1016/0165-3806(86)90293-2

[56] Lorenc-Duda A, Berezińska M, Urbańska A, Gołembiowska K, Zawilska JB (2009) Dopamine in the Turkey retina-an impact of environmental light, circadian clock, and melatonin. J Mol Neurosci 38: 12–18. 18953673 10.1007/s12031-008-9153-8

[57] Shelke RR, Lakshmana MK, Ramamohan Y, Raju TR (1997) Levels of dopamine and noradrenaline in the developing of retina – effect of light deprivation. Int J Dev Neurosci 15: 139–143. 9099624 10.1016/s0736-5748(96)00080-9

[58] Spira AW, Parkinson D (1991) Effects of dark-rearing on the retinal dopaminergic system in the neonatal and postnatal guinea pig. Brain Res Dev Brain Res 62: 142–145. 1760868 10.1016/0165-3806(91)90200-3

[59] Huppé-Gourgues F, Coudé G, Lachapelle P, Casanova C (2005) Effects of the intravitreal administration of dopaminergic ligands on the b-wave amplitude of the rabbit electroretinogram. Vis Res 45: 137–145. 15581915 10.1016/j.visres.2004.08.001

[60] Kim DY, Jung CS (2012) Gap junction contributions to the goldfish electroretinogram at the photopic illumination level. Korean J Physiol Pharmacol 16: 219–224. 22802705 10.4196/kjpp.2012.16.3.219 PMC3394926

[61] Jackson CR, Ruan GX, Aseem F, Abey J, Gamble K, et al (2012) Retinal dopamine mediates multiple dimensions of light-adapted vision. J Neurosci 32: 9359–9368. 22764243 10.1523/JNEUROSCI.0711-12.2012 PMC3400466

[62] Lavoie J, Illiano P, Sotnikova TD, Gainetdinov RR, Beaulieu JM, et al.. (2013) The Electroretinogram as a Biomarker of Central Dopamine and Serotonin: Potential Relevance to Psychiatric Disorders. Biol Psychiatry S0006–3223(12)01032–01033. 10.1016/j.biopsych.2012.11.024 23305992

[63] Popova E, Kupenova P (2011) Effects of dopamine D (1) receptor blockade on the intensity-response function of ERG b- and d-waves under different conditions of light adaptation. Vis Res 51: 1627–1636. 21605587 10.1016/j.visres.2011.05.005

[64] Stockton RA, Slaughter MM (1989) B-wave of the electroretinogram. A reflection of ON bipolar cell activity. J Gen Physiol 93: 101–122. 2915211 10.1085/jgp.93.1.101 PMC2216200

[65] Tian N, Slaughter MM (1995) Correlation of dynamic responses in the ON bipolar neuron and the b-wave of the electroretinogram. Vis Res 35: 1359–1364. 7645264 10.1016/0042-6989(95)98715-l

[66] Wachtmeister L (1998) Oscillatory potentials in the retina: what do they reveal. Prog Retin Eye Res 17: 485–521. 9777648 10.1016/s1350-9462(98)00006-8

[67] Severns ML, Johnson MA, Bresnick GH (1994) Methodologic dependence of electroretinogram oscillatory potential amplitudes Doc Ophthalmol. 86: 23–31. 10.1007/BF01224625 7956683

[68] Rodriguez-Saez E, Otero-Costas J, Moreno-Montañes J, Relova JL (1993) Electroretinographic changes during childhood and adolescence. Eur J Ophthalmol 3: 6–12. 8485400 10.1177/112067219300300102

[69] el Azazi M, Wachtmeister L (1991) The postnatal development of the oscillatory potentials of the electroretinogram. III. Scotopic characteristics. Acta Ophthalmol (Copenh) 69: 505–510. 1750319 10.1111/j.1755-3768.1991.tb02029.x

[70] el Azazi M, Wachtmeister L (1991) The postnatal development of the oscillatory potentials of the electroretinogram. II. Photopic characteristics. Acta Ophthalmol (Copenh) 69: 6–10. 2028769 10.1111/j.1755-3768.1991.tb01983.x

[71] Baxter BI, Riesen AH (1961) Electroretinogram of the visually deprived cat. Science 134: 1626–1627. 13866003 10.1126/science.134.3490.1626

[72] Babkoff H (1975) The effect of light deprivation on the B-wave input-output function. Ann Ophthalmol 7: 1335–1338. 1190659

[73] Babkoff H (1977) Light-deprivation and light-adaptation: a preliminary study. Ann Ophthalmol 9: 1535–1539. 606034

[74] Saszik S, Bilotta J (2001) Constant dark-rearing effects on visual adaptation of the zebrafish ERG. Int J Dev Neurosci 19: 611–619. 11705665 10.1016/s0736-5748(01)00051-x

[75] Holopigian K, Clewner L, Seiple W, Kupersmith MJ (1994) The effects of dopamine blockade on the human flash electroretinogram. Doc Ophthalmol 86: 1–10. 7956681 10.1007/BF01224623

[76] Schneider T, Zrenner E (1991) Effects of D-1 and D-2 dopamine antagonists on ERG and optic nerve response of the cat. Exp Eye Res 52: 425–430. 2037020 10.1016/0014-4835(91)90038-g

[77] Oliver P, Jolicoeur FB, Lafond G, Drumheller A, Brunette JR (1987) Effects of retinal dopamine depletion on the rabbit electroretinogram. Doc Ophthalmol 66: 359–371. 3123188 10.1007/BF00213664

[78] Wachtmeister L, Dowling JE (1978) The oscillatory potentials of the mudpuppy retina. Invest Ophthalmol Vis Sci 17: 1176–1188. 721390

[79] Kobayashi A, Shirao Y, Tagawa S, Katoh K, Tamura T (1996) Effects of retinal intrinsic dopamine on the in vivo electroretinogram of rabbits. Nihon Ganka Gakkai Zasshi 100: 111–117. 8851149

[80] Bloomfield SA, Völgyi B (2009) The diverse functional roles and regulation of neuronal gap junctions in the retina. Nat Rev Neurosci 10: 495–506. 19491906 10.1038/nrn2636 PMC3381350

[81] Xin D, Bloomfield SA (1999) Dark- and light-induced changes in coupling between horizontal cells in mammalian retina. J Comp Neurol 405: 75–87. 10022197 10.1002/(sici)1096-9861(19990301)405:1<75::aid-cne6>3.0.co;2-d

[82] Baldridge WH, Weiler R, Dowling JE (1995) Dark suppression and light-sensitization of horizontal cell responses in the hybrid bass retina. Vis Neurosci 12: 611–620. 8527363 10.1017/s0952523800008907

[83] Bloomfield SA, Xin D, Osborne T (1997) Light-induced modulation of coupling between AII amacrine cells in the rabbit retina. Vis Neurosci 14: 565–576. 9194323 10.1017/s0952523800012220

[84] Xin D, Bloomfield SA (1999) Comparison of the responses of AII amacrine cells in the dark- and light adapted rabbit retina. Vis Neurosci 16: 653–665. 10431914 10.1017/s0952523899164058

[85] Lasater EM (1987) Retinal horizontal cell gap junctional conductance is modulated by dopamine through a cyclic AMP-dependent protein kinase. Proc Natl Acad Sci USA 84: 7319–7323. 2823257 10.1073/pnas.84.20.7319 PMC299284

[86] DeVries SH, Schwartz EA (1989) Modulation of an electrical synapse between solitary pairs of catfish horizontal cells by dopamine and second messengers. J Physiol 414: 351–375. 2558170 10.1113/jphysiol.1989.sp017692 PMC1189146

[87] Kothmann WW, Massey SC, O'Brien J (2008) Dopamine D1-receptor-mediated modulation of connexin36 phosphorylation in AII amacrine cells. Invest Ophthal Vis Sci 49: 1515.

[88] Mills SL. Massey SC (1995) Differential properties of two gap junctional pathways made by AII amacrine cells. Nature 377: 734–737. 7477263 10.1038/377734a0

[89] Urschel S, Höher T, Schubert T, Alev C, Söhl G, et al (2006) Protein kinase A-mediated phosphorylation of connexin36 in mouse retina results in decreased gap junctional communication between AII amacrine cells. J Biol Chem 281: 33163–33171. 16956882 10.1074/jbc.M606396200

[90] Völgyi B, Deans MR, Paul DL, Bloomfield SA (2004) Convergence and segregation of the multiple rod pathways in mammalian retina. J Neurosci 24: 11182–11192. 15590935 10.1523/JNEUROSCI.3096-04.2004 PMC2834589

[91] Kirsch M, Wagner HJ, Djamgoz MB (1991) Dopamine and plasticity of horizontal cell function in the teleost retina: regulation of a spectral mechanism through D1-receptors. Vis Res 31: 401–412. 1843751 10.1016/0042-6989(91)90093-k

[92] Pfeiffer-Linn C, Lasater EM (1993) Dopamine modulates in a differential fashion T- and L-type calcium currents in bass retinal horizontal cells. J Gen Physiol 102: 277–294. 8228912 10.1085/jgp.102.2.277 PMC2229148

[93] Wagner HJ, Djamgoz MB (1993) Spinules: a case for retinal synaptic plasticity. Trends Neurosci 16: 201–206. 7688159 10.1016/0166-2236(93)90155-f

[94] Bui BV, Vingrys AJ (1999) Development of receptoral responses in pigmented and albino guinea-pigs (Cavia porcellus). Doc Ophthalmo 99: 151–170. 10.1023/a:1002721315955 11097119

[95] Braekevelt CR, Hollenberg MJ (1970) The development of the retina of the albino rat. Am J Anat 127: 281–301. 5436824 10.1002/aja.1001270305

[96] Masland RH (1977) Maturation of function in the developing rabbit retina. J Comp Neurol 175: 275–286. 903424 10.1002/cne.901750303

[97] Weidman TA, Kuwabara T (1968) Postnatal development of the rat retina. An electron microscopic study. Arch Ophthal 79: 470–484. 5640327 10.1001/archopht.1968.03850040472015

[98] Weidman TA, Kuwabara T (1969) Development of the rat retina. Invest Ophthalmol 8: 60–69. 5763846

[99] Hamasaki DI, Maguire GW (1985) Physiological development of the kitten's retina: an ERG study. Vis Res 25: 1537–1543. 3832577 10.1016/0042-6989(85)90124-5

[100] Tucker GS, Hamasaki DI, Labbie A, Bradford N (1982) Physiologic and anatomic development of the photoreceptors of normally-reared and dark-reared rabbits. Expert Brain Res 48: 263–271. 10.1007/BF00237222 7173362

[101] Kramer SG (1971) Dopamine: A retinal neurotransmitter. I. Retinal uptake, storage, and light-stimulated release of H3-dopamine in vivo. Invest Ophthalmol 10: 438–452. 4325307

[102] Kirsch M, Wagner HJ (1989) Release pattern of endogenous dopamine in teleost retinae during light adaptation and pharmacological stimulation. Vision Res 29: 147–154. 2800344 10.1016/0042-6989(89)90120-x

[103] Godley BF, Wurtman RJ (1988) Release of endogenous dopamine from the superfused rabbit retina in vitro: effect of light stimulation. Brain Res 452: 393–395. 3401747 10.1016/0006-8993(88)90046-7

[104] Weiler R, Baldridge WH, Mangel SC, Dowling JE (1997) Modulation of endogenous dopamine release in the fish retina by light and prolonged darkness. Vis Neurosci 14: 351–356. 9147486 10.1017/s0952523800011470

[105] Miranda-Anaya M, Bartell PA, Menaker M (2002) Circadian rhythm of iguana electroretinogram: the role of dopamine and melatonin. J Biol Rhythms 17: 526–538. 12465886 10.1177/0748730402238235

[106] Nir I, Harrison JM, Haque R, Low MJ, Grandy DK, et al (2002) Dysfunctional light-evoked regulation of cAMP in photoreceptors and abnormal retinal adaptation in mice lacking dopamine D4 receptors. J Neurosci 22: 2063–2073. 11896146 10.1523/JNEUROSCI.22-06-02063.2002 PMC6758276

[107] Seki M, Nawa H, Fukuchi T, Abe H, Takei N (2003) BDNF is upregulated by postnatal development and visual experience: quantitative and immunohistochemical analyses of BDNF in the rat retina. Invest Ophthalmol Vis Sci 44: 3211–3218. 12824273 10.1167/iovs.02-1089

[108] Xu HP, Tian N (2007) Retinal ganglion cell dendrites undergo a visual activity-dependent redistribution after eye-opening. J Comp Neurol 503: 244–259. 17492624 10.1002/cne.21379

[109] Di Marco S, Nguyen VA, Bisti S, Protti DA (2009) Permanent functional reorganization of retinal circuits induced by early long-term visual deprivation. J Neurosci 29: 13691–13701. 19864581 10.1523/JNEUROSCI.3854-09.2009 PMC6665001

[110] Sherman SM, Stone J (1973) Physiological normality of the retinal in visually deprived cats. Brain Res 60: 224–230. 4744763 10.1016/0006-8993(73)90861-5

[111] Wiesel TN, Hubel DH (1963) Effects of visual deprivation on the morphology and physiology of cells in the cat's lateral geniculate body. J Neurophysiol 26: 978–993. 14084170 10.1152/jn.1963.26.6.978

[112] Kratz KE, Mangel SC, Lehmkuhle S, Sherman M (1979) Retinal X- and Y-cells in monocularly lid-sutured cats: normality of spatial and temporal properties. Brain Res 172: 545–551. 476497 10.1016/0006-8993(79)90586-9

[113] He Q, Wang P, Tian N (2011) Light-evoked synaptic activity of retinal ganglion and amacrine cells is regulated in developing mouse retina. Eur J Neurosci 33: 36–48. 21091802 10.1111/j.1460-9568.2010.07484.x PMC3070459

