# Correction: Dopamine D1 Receptors Regulate the Light Dependent Development of Retinal Synaptic Responses

**DOI:** 10.1371/journal.pone.0100048

**Published:** 2014-06-06

**Authors:** The PLOS ONE Staff

There are errors in the funding statement. The correct funding statement is as follows: This work was supported by NIH grant R01EY012345, Research to Prevent Blindness (RPB) and Fight for Sight (He, PD02035). The funders had no role in study design, data collection and analysis, decision to publish, or preparation of the manuscript.
